# *Aedes aegypti* continuously exposed to *Bacillus thuringiensis* svar. *israelensis* does not exhibit changes in life traits but displays increased susceptibility for Zika virus

**DOI:** 10.1186/s13071-021-04880-6

**Published:** 2021-07-28

**Authors:** Karine da Silva Carvalho, Duschinka Ribeiro Duarte Guedes, Mônica Maria Crespo, Maria Alice Varjal de Melo-Santos, Maria Helena Neves Lobo Silva-Filha

**Affiliations:** Department of Entomology, Instituto Aggeu Magalhães-Fiocruz, Recife, Pernambuco Brazil

**Keywords:** Fitness, DENV, Artificial infection, Infection, Vector competence

## Abstract

**Background:**

*Aedes aegypti* can transmit arboviruses worldwide, and *Bacillus thuringiensis* svar. *israelensis* (Bti)-based larvicides represent an effective tool for controlling this species. The safety of Bti and lack of resistance have been widely reported; however, little is known regarding the impact of the extensive use of these larvicides on the life traits of mosquitoes. Therefore, this study investigated biological parameters, including susceptibility to arbovirus, of an *Ae. aegypti* strain (RecBti) subjected to 29 generations of exposure to Bti compared with the RecL reference strain.

**Methods:**

The biological parameters of individuals reared under controlled conditions were compared. Also, the viral susceptibility of females not exposed to Bti during their larval stage was analysed by oral infection and followed until 14 or 21 days post-infection (dpi).

**Results:**

RecBti individuals did not display alterations in the traits that were assessed (fecundity, fertility, pupal weight, developmental time, emergence rate, sex ratio and haematophagic capacity) compared to RecL individuals. Females from both strains were susceptible to dengue serotype 2 (DENV-2) and Zika virus (ZIKV). However, RecBti females showed significantly higher rates of ZIKV infection compared with RecL females at 7 (90% versus 68%, Chi-square: χ^2^ = 7.27, *df* = 1, *P* = 0.006) and 14 dpi (100% versus 87%, Chi-square: χ^2^ = 7.69, *df* = 1, *P* = 0.005) and for dissemination at 7 dpi (83.3% versus 36%, Fisher’s exact test: *P* < 0.0001, OR = 0.11, 95% CI 0.03–0.32). Quantification of DENV-2 and ZIKV viral particles produced statistically similar results for females from both strains.

**Conclusions:**

Prolonged exposure of *Ae. aegypti* larvae to Bti did not alter most of the evaluated biological parameters, except that RecBti females exhibited a higher vector susceptibility for ZIKV. This finding is related to a background of Bti exposure for several generations but not to a previous exposure of the tested females during the larval stage. This study highlights mosquito responses that could be associated with the chronic exposure to Bti in addition to the primary larvicidal effect elicited by this control agent.

**Graphical abstract:**

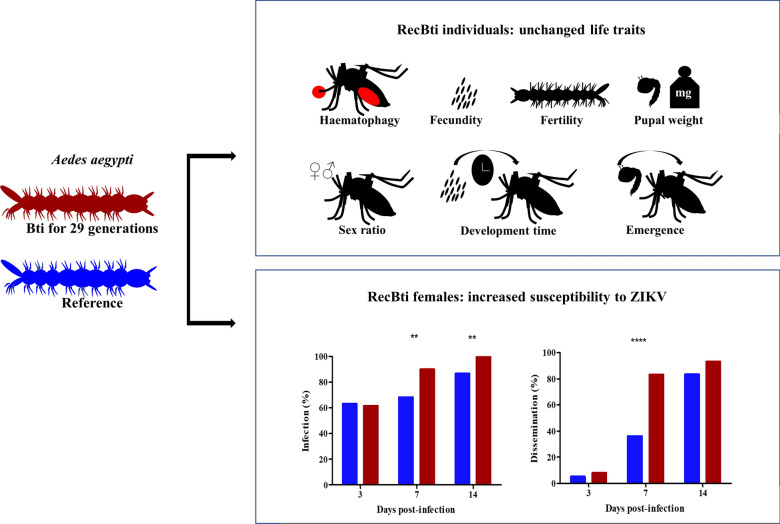

**Supplementary Information:**

The online version contains supplementary material available at 10.1186/s13071-021-04880-6.

## Background

Infections caused by arbovirus dengue (DENV), chikungunya (CHIKV) and Zika (ZIKV) are global public health threats, and their transmission to humans relies primarily on *Aedes aegypti* and *Aedes albopictus* mosquitoes [1, 2]. Brazil is among the most severely affected countries and is hyperendemic for all DENV, and the introduction of CHIKV and ZIKV has amplified the burden caused by arboviruses in this country [3]. The major approach for interrupting the transmission of these diseases relies on vector control, as effective and accessible vaccines and therapeutic drugs are not available to date [4]. Controlling *Aedes* spp. has been a challenge, since these species display outstanding survival strategies that enable them to occupy, spread and establish in the environment successfully [5–8]. Currently, emerging and conventional control methods can be integrated to fight *Aedes*, and the use of larvicides remains an important intervention that can be adopted in this context [9, 10]. These include environmentally safe products with specific modes of action, such as *Bacillus thuringiensis* svar. *israelensis* (Bti)-based larvicides [11]. The active principle of these larvicides is an insecticidal crystal produced by Bti that contains four major protoxins (Cry11Aa, Cry4Aa, Cry4Ba and Cyt1Aa) that kill mosquito larvae [11]. Bti crystals act by ingestion and, after they are solubilized in the midgut, protoxins are released and processed into active toxins that interact with midgut receptors. After the toxins specifically bind to receptors, they cause pore formation and are internalized in the cells, leading to osmotic lysis and larval death [12, 13]. Bti crystals display high larvicidal activity, but they have a selective spectrum, since they target only certain Diptera species, including those from the *Aedes* genus [11]. The three-domain Cry-type toxins from Bti (Cry11Aa, Cry4Ba, and Cry4Aa) can specifically bind to different membrane-bound receptors, such as cadherins, aminopeptidases and alkaline phosphatases that have been identified in *Ae. aegypti* larvae [12, 14, 15]. On the other hand, Cyt1Aa is a cytolytic toxin with the intrinsic ability to insert itself into the cell membrane and form pores [16, 17], and this toxin plays an important role in the mode of action of the Bti crystal. Cyt1Aa can act as a receptor for the other Cry toxins, binding and promoting their oligomerization, which enables them to bind to their midgut receptors with a higher affinity and form pores on the cell membrane [18–20]. The ability of Cyt1Aa to synergize with Cry toxins considerably reduces the selection of resistance caused by receptor alteration, which is the most common resistance mechanism reported for *Bacillus thuringiensis* toxins [21, 22].

Therefore, Bti is one of the most effective recommended larvicides for controlling *Aedes* spp. worldwide in view of its complex and safe mode of action, having been used over the last 4 decades [5, 7, 23–26]. Due to safety issues, only a few larvicides, including Bti larvicides, remain authorized for mosquito control according to the legislation of the European Union [27, 28]. In view of the increase in Bti utilization, the impact of the continuous exposure of mosquito populations needs to be assessed, particularly considering the scenario of endemic countries that conduct vector control actions throughout the year [29]. To date, most investigations have focused on resistance and environmental safety. These studies have indicated the absence of resistance to the Bti crystal [25, 26, 29–33] and showed its safety over almost those decades of use [34–36]. Notably, resistance to individual toxins has been reported using laboratory selection procedures but not resistance to the whole crystal, which is the active principle of Bti-based products [37–40].

Another important aspect, the influence of Bti exposure on the life traits of target insects, has only rarely been investigated. Most studies have investigated the biological cost associated with mosquitoes that are resistant to chemical insecticides [41–43] and the vector competence that could affect their capacity to become infected and transmit pathogens to humans [44–46]. Our hypothesis is that, although continuous exposure to Bti did not elicit the selection of resistance, other parameters of vector biology could be modified in response to this condition and should be investigated to understand the consequences of the chronic exposure to this larvicide. The major goal of this study was to investigate whether an *Ae. aegypti* strain continuously exposed to Bti for 29 generations displays changes in life traits, such as fecundity, fertility, pupal weight, developmental time, emergence rate, sex ratio, haematophagic capacity and vector susceptibility for DENV and ZIKV. This local strain, RecBti, was first established in our laboratory to evaluate the selection of Bti resistance after exposing larvae to Bti for 30 generations under controlled conditions [33]. No resistance to Bti was detected, and RecBti larvae were also susceptible to other insecticidal compounds, such as temephos and diflubenzuron. Similarly, the activity of the detoxifying enzymes involved in the metabolism of insecticidal compounds was unchanged. Taken together, these findings indicate that Bti use is compatible with other classes of insecticides with a low risk of resistance to Bti itself and of cross-resistance to other control agents [33].

## Methods

### *Aedes aegypti* strains

Two *Ae. aegypti* strains were used in this study, RecL and RecBti, which were maintained at the insectary of the Instituto Aggeu Magalhães (IAM)-FIOCRUZ at 26 ± 1 °C, 70% humidity and a 14 h:10 h light:dark photoperiod. Larvae were reared in dechlorinated tap water and fed cat food (Friskies^®^). Adults were fed a sucrose solution (10%), and females were also artificially fed defibrinated rabbit blood once per week. RecL and RecBti strains were founded using larvae from Recife city; therefore, they share the same geographic origin. RecL is a local reference strain that has been maintained in the insectary without contact with any mosquito control agent since 1996 [47]. The test strain RecBti was established in 2011, and larvae from each generation have been continuously exposed to a Bti-based larvicide for more than 30 generations and selection procedures were described in our previous study [33]. The selection pressure imposed on the large samples of individuals from the RecBti strain, originated from Recife as the RecL strain, was strong and continuous using a commercial Bti-based product containing 37.4% Bti crystals/spores as active ingredient. Briefly, around 9,500 third-instar larvae from every generation were treated with Bti, and around 74% mortality was detected during the pre-imaginal phase. At least 2,000 adults that survived the Bti exposure per generation were used to compose the next parental generation. Bti exposure was carried during 30 generations and involved > 290,000 larvae, as fully described by Carvalho et al. [33]. Susceptibility bioassays performed using larvae from generations F_5_ to F_30_ demonstrated they were susceptible to Bti, despite continuous exposure to this agent, as shown by resistance ratios (RR at LC_50_): 1.6 (F_1_), 2.8 (F_5_), 2.3 (F_10_), 1.5 (F_15_), 1.1 (F_20_), 0.9 (F_25_) and 1.5 at F_30_ [33]. Individuals of the F_30_ generation were employed for this study and the last Bti exposure was performed on F_29_ third-instar larvae. Therefore, F_30_ larvae were not treated with Bti before adult emergence because the goal of this study was to record the chronic response of the colony and not the induced response to a recent Bti exposure during the immature stage of the individuals analysed.

### Mosquito maintenance for biological assessment

To assess such biological parameters as fecundity, fertility, pupal weight, developmental time, emergence rate, sex ratio and haematophagic capacity, individuals from both strains were kept in the insectary under controlled rearing parameters, as described in this section. First, filter papers containing stored eggs were subject to induced eclosion by setting them in a recipient with grass infusion (6 g/l) for 24 h. Samples of 100 first-instar larvae, collected within 24 h after eclosion, were transferred to plastic recipients (20 cm length × 16 cm width × 8 cm deep, 3-l capacity) with 1 l tap water and fed 350 mg of cat food (Friskas^®^) provided on days 0 (100 mg), 4 (150 mg) and 5 (100 mg) during the assays. After emergence, samples of 20 females and 20 males (1:1 ratio) were set in a plastic container (12 cm height × 10 cm diameter, 2.3-l capacity) and fed sucrose solution (10%) *ad libitum*. Five days post-emergence, a single meal of defibrinated rabbit blood was offered to females using an artificial feeding system for 1 h at 37 °C. This system consisted of a blood sample (8 ml) set in a Petri dish (6 cm height × 1.5 cm diameter) covered by a double layer of Parafilm^®^ membrane, placed on each mosquito cage, and kept at 37 °C using heat packs. Immediately after the blood meal, only engorged females, selected by visual abdomen inspection, were transferred to cages and fed sucrose solution (10%) *ad libitum*. Three days after the blood meal, two recipients each with water and two sections of filter paper (7 cm length × 5 cm width) were placed in cages as a substrate for female oviposition for two days. After that step, filter papers containing eggs were collected and allowed to dry at insectary room temperature to complete embryonic development. After four days of storage, eggs were counted, subjected to eclosion and reared, as described above.

### Assessment of life traits

The life traits of RecBti (F_30_) and RecL individuals reared according to the procedures described in the previous section were compared. For each parameter analysed, three replicates per colony were tested, and three independent experiments were carried out. Fecundity was determined as the total number of eggs recorded on the filter papers available for the oviposition of a group of 20 engorged females. Fertility was recorded as the total number of first-instar larvae that hatched from a sample of 2,551 eggs that were subjected to the protocol of eclosion for 24 h. Pupal weight was determined by measuring pools of 25 living pupae of each sex. For this purpose, pupae were collected until 14 h after the moult, water was drained, and samples were weighed and immediately placed back in their rearing containers, where they were kept until emergence. Adults obtained from these samples were used to evaluate adult rate, immature developmental time, sex ratio and haematophagic capacity, as described below. The adult rate was determined from a sample of 900 first-instar larvae that reached emergence within 15 days. The sex ratio among these males and females was also recorded. The haematophagic capacity was determined by the percentage of engorged females after the artificial blood meal from the pools of 20 females per cage.

### Viral strains and sample preparation for vector susceptibility assays

The susceptibility of RecBti females to dengue serotype 2 (DENV-2) and Zika (ZIKV) viruses was investigated and compared to RecL females based on three independent artificial blood-feeding assays. The viral stocks used in this study were generously provided by the Laboratory of Virology and Experimental Therapy from IAM-Fiocruz. DENV-2 strain 3808/BR-PE and ZIKV strain 243/BR-PE were isolated from patients during dengue [48] and Zika [49] outbreaks in Recife in 1995 and 2015, respectively. These strains are able to infect and disseminate in RecL females as previously described [50, 51]. Viral stocks were then produced in C6/36 cells after five passages for DENV-2 and in VERO cells for ZIKV after four passages, and both were stored at − 80 °C until use. Prior to artificial blood-feeding experiments, DENV-2 viral stock was grown in C6/36 cells at a multiplicity of infection of 0.1 for 5–6 days, and ZIKV viral stock was grown in VERO cells at a multiplicity of infection of 0.5 for 4–5 days, when the cytopathic effects were visualized, which allowed the use of those samples for the infection assays [50, 51]. Noninfected cell cultures were maintained under the same conditions. The mean virus titration for the three assays based on the Tissue culture infection dose at 50% (TCID_50_) for DENV-2 was 4.19 × 10^5^ TCID_50_/ml while the determination by assay plaque for ZIKV was 1.37 × 10^6^ plaque-forming units, (PFU)/ml.

### Oral artificial infection blood-feeding procedure

Each experiment was carried out using nulliparous 7- to 10-day-old females per strain starved for 24 h. A sample between 110 and 130 females from each strain was set in a cage (12 cm height × 10 cm diameter, 2.3-l capacity) and fed defibrinated rabbit blood containing cultures infected with DENV-2 or ZIKV. Another sample of 30 females per strain, set in another cage, was fed defibrinated rabbit blood containing uninfected cell cultures and used as the untreated control group. To prepare the blood meal, cell culture samples were subjected to a single cycle of freezing and thawing to lyse the cells releasing viral particles, allowing the use of comparable and reproducible titles [52]. To this end, cell culture flasks were kept at − 80 °C for only 11 min, thawed in running water and then mixed with defibrinated rabbit blood in a 1:1 ratio. These samples were immediately used for feeding all experimental groups simultaneously, and the virus suspensions were also quantified, as described in the previous section. After feeding, only engorged females, selected by visual abdomen inspection, were transferred to another cage. These females were maintained under standard conditions for 21 days for DENV-2 and 14 days for ZIKV experiments, as previously described [50, 51].

### RNA extraction and virus detection

RNA was extracted from each head and body part from individual females per experimental point (*n* = 20 females/point), and each sample was analysed in duplicate [50, 51]. For DENV-2 infection assays, females were collected just after feeding (0) and at 7, 14 and 21 day(s) post-infection (dpi) [51]. For ZIKV assays, females were collected at 0, 3, 7 and 14 dpi [50, 53]. Females fed uninfected blood were collected just after the feeding procedure (*n* = 5). Females were collected and killed in 70% alcohol at 2 °C for 2 s. Samples were washed twice in ultrapure water at 2 °C. Next, the body and head (with attached salivary glands) from each female were carefully dissected on a cold plate (2 °C). The dissected parts were immediately placed in separate DNase/RNase-free microtubes at 2 °C with 300 μl of mosquito diluent containing 0.1 M phosphate-buffered saline-PBS (Na_2_HPO_4_ 7.7 mM, K_2_HPO_4_ 1.1 mM, KCl 2.7 mM, NaCl 137 mM, pH 7.4) and supplemented with 10% foetal bovine serum (FBS) and 1% Fungizone^®^ (Gibco #15290-018) [54]. Samples were stored at − 80 °C until RNA extraction. Total RNA extraction of the tissue homogenate (100 μl) was performed using Trizol^®^ following the manufacturer’s protocol (Invitrogen #15596-026) with modifications as described in Guedes et al. [50]. Samples were further treated with Turbo DNase^®^ (Ambion #AM2239) to prevent DNA contamination and stored at − 80 °C. Virus detection and quantification in the samples were performed by quantitative real-time polymerase chain reaction (RT-qPCR) using an ABI Prism 7500 SDS Real-Time system^®^ (Applied BioSystems). RT-qPCR assays for DENV-2 detection were performed using a SYBR Green RT-PCR Kit (QIAGEN #204245) according to previous protocols. The reaction contained the RNA sample (5 μl with an average concentration of 180 ng/μl), SYBR Green Master^®^ mix 1X (10 μl), primers (0.2 μM) based on Kong et al. [55] (Additional file 1: Table S1), reverse transcriptase (0.2 μl) and ultrapure water for a 20-μl final volume. Reaction cycling conditions were as follows: 50 °C for 30 min; 95 °C for 15 min to activate Taq; 40 cycles of 94 °C for 15 s, 58 °C for 30 s and 72 °C for 30 s [51]. For ZIKV, those assays were performed using a QuantiNova Probe RT-PCR kit (QIAGEN #208352), and the reaction mix contained the following: the RNA sample (5 μl with an average concentration of 180 ng/μl), QuantiNova Probe RT-PCR Master Mix (2X, 20 μl), QuantiNova Probe RT Mix (0.2 μl), ROX Reference Dye (0.1 μl) and primers [56] (100 μM) (Additional file 1: Table S1) in a 20-μl final volume. RT-qPCR cycling included a single cycle of reverse transcription for 15 min at 45 °C followed by 5 min at 95 °C and then 45 cycles of 5 s at 95 °C and 45 s at 60 °C [50]. The viral RNA quantification of samples used for the assays was determined by absolute quantification using cycle threshold (Ct) values from a standard curve of known concentrations using a ten-fold dilution (10^–2^ to 10^–6^) of purified viral transcripts included in each PCR plate [55]. Positive control samples were RNA extracted from infected culture supernatant, and negative control samples were RNA extracted from uninfected culture supernatant and samples of RT-qPCR reaction mix without RNA. Samples with a melting curve for the specificity of the amplified products (~ 78.6 °C) were considered positive for DENV-2 RNA. Positive samples for ZIKV RNA were those with a Ct value ≤ 38.5. The amount of DENV-2 and ZIKV viral RNA in each sample was calculated based on the Ct values from the standard curve of the viral RNA included in each PCR plate.

### Statistical analysis

Life traits from the two strains were statistically analysed by Student’s t-test, with *P* < 0.05 being considered significant. For virus susceptibility assays, two parameters were analysed based on the positive samples for the presence of virus RNA. The infection rate (IR) was calculated by the number of positive body samples divided by the total number of mosquitoes analysed. The disseminated infection rate (DIR) is the proportion of infected head samples considering the total number of infected body samples [57]. For statistical analysis, the Chi-square and Fisher’s test were used to evaluate the IR and DIR between the two strains tested. To compare the number of RNA viral copies, the Kruskal-Wallis and Mann-Whitney tests were used, considering a *P*-value < 0.05 to be significant. The percentage of females blood-fed and non-fed of cells with uninfected or infected cultures were statistically analysed by Student’s t-test, with *P* < 0.05 being considered to be significant.

## Results

### Life traits

To investigate whether the continuous exposure of *Ae. aegypti* larvae to Bti larvicide for 29 generations affects their biological performance, seven life traits from RecBti individuals were assessed and compared to those of the RecL reference strain (Table 1). The fecundity of RecBti females, based on > 8,988 eggs from a pool of 20 females, showed a similar average of 93.2 eggs/female compared with 86.5 eggs/female from RecL females (*t*_(4)_ = 2.77, *P* = 0.432). A mean of 90.6% of eggs produced by RecBti females was viable, which was statistically similar to those of RecL females and indicated successful insemination. The pupal weight of a pool of 25 RecBti specimens weighed 69.1 mg and 122.2 mg on average for male and female pools, respectively, and similar measurements were found for RecL pupal pools. Considering the time of development of first-instar larvae to the adult phase, we found that emergence took an average of 10.4 and 11.1 days for RecBti and RecL individuals, respectively, which were statistically similar (*t*_(4)_ = 2.77, *P* = 0.093). From a sample of individuals analysed, the adult rate was notably high (96–97%); therefore, most first-instar larvae from both strains achieved emergence. The sex ratio recorded using samples of > 800 adults per strain showed that an equal proportion of males and females (1.1: 0.9) was produced by both strains. The haematophagic capacity of RecBti females was lower (80%) than that of RecL (89.2%) females, but this reduction was not significantly different (*t*_(4)_ = 2.77, *P* = 0.170). The rates of blood-fed females from both mosquito strains shown in the next sections are in keeping with these findings, except for females blood-fed and non-fed from assays DENV-2 with cell culture uninfected (*t*_(4)_ = 2.77, *P* = 0.033) (Additional file 2: Table S2). The parameters analysed in this section indicate that RecBti individuals exhibited characteristics similar to those of individuals from a reference colony.

**Table 1:** Life traits of *Aedes aegypti* from RecBti and RecL strains

Parameters	RecBti		RecL		Student’s t-test^a^
	*n*	Mean ± SD	*n*	Mean ± SD	
Fecundity (mean no. eggs/female)	8988^b^	93.2 ± 3.3	8463^b^	86.5 ± 7.6	*t*_(4)_ = 2.77, *P* = 0.43
Fertility (% first-instar larvae)	2551	90.6 ± 2.8	2526	89.9 ± 3.7	*t*_(4)_ = 2.77, *P* = 0.79
Pupal weight (mg), per pool of 25 males	16	69.1 ± 3.4	16	67.3 ± 3.9	*t*_(4)_ = 2.77, *P* = 0.15
Pupal weight (mg), per pool of 25 females	13	122.2 ± 7.4	12	121 ± 4.5	*t*_(4)_ = 2.77, *P* = 0.61
Development time (days)^c^	864	10.4 ± 0.5	873	11.1 ± 1.1	*t*_(4)_ = 2.77, *P* = 0.09
Adult rate (%)	900	96 ± 2.2	900	97 ± 2.1	*t*_(4)_ = 2.77, *P* = 0.77
Sex ratio (male:female)	864	1.1:0.9 ± 0.1	873	1.1:0.9	*t*_(4)_ = 2.77, *P* = 0.74
Haematophagic rate (% blood-fed)	120	80 ± 12.6	120	89.2 ± 8.0	*t*_(4)_ = 2.77, *P* = 0.17

^a^For *P* ≥ 0.05, the mean of each parameter was not significantly different according to Student’s t-test

^b^Number of 8988 and 8463 eggs laid by 96 and 108 blood-fed females, respectively

^c^Period from first-instar larvae to emergence

### Mosquito susceptibility to DENV-2

**Fig. 1:**
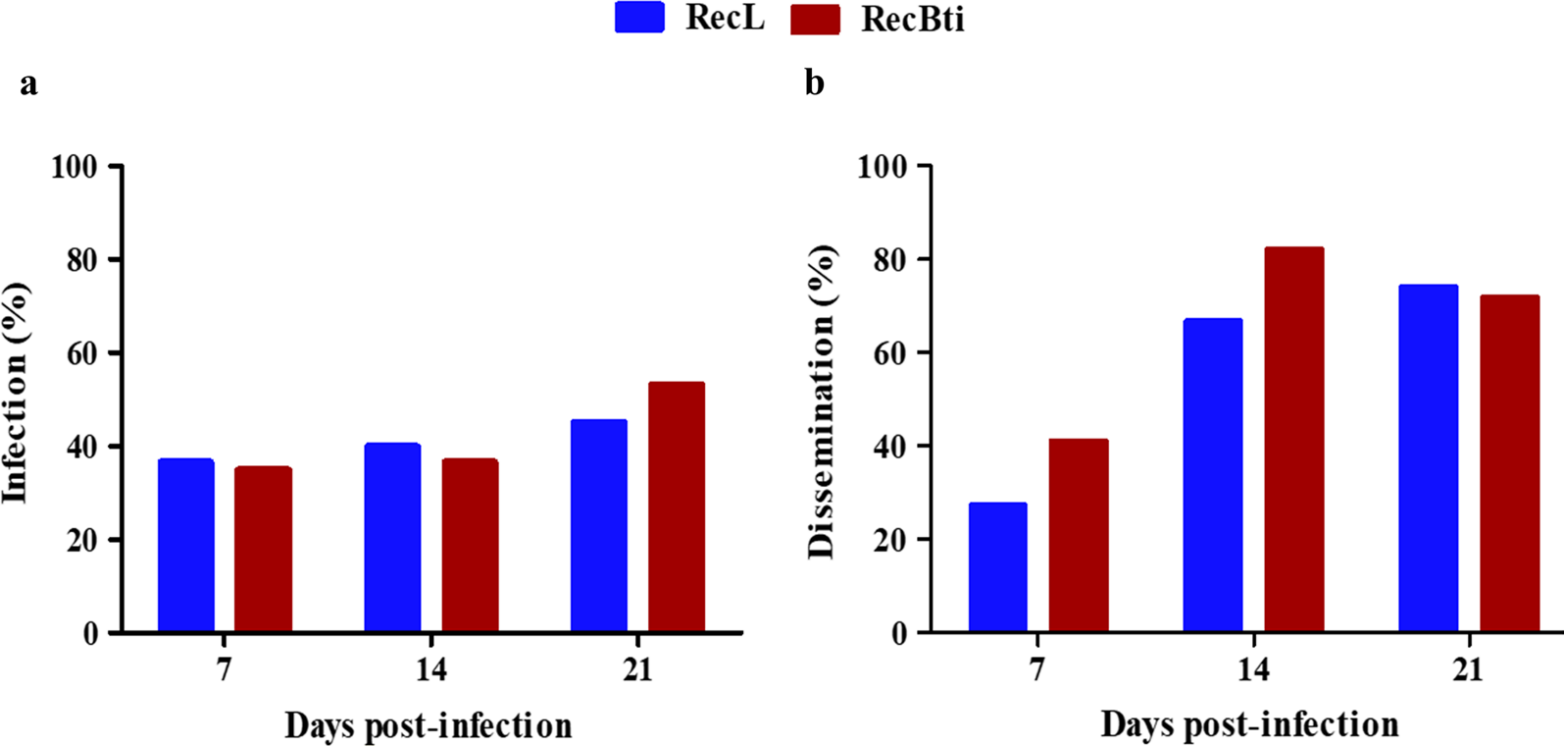
Detection of DENV-2 RNA in *Aedes aegypti* females from RecBti and RecL strains. **a** Infection rate. **b** Dissemination rate. Each experimental point was based on a sample of 60 individuals from three independent assays. Each column represents positives samples in absolute numbers. According to Chi-square and Fisher’s exact tests, the percentage between mosquito strains is not significantly different for *P* ≥ 0.05

To investigate whether exposure to Bti would have an impact on the susceptibility of the RecBti strain to DENV-2, blood-fed females were subjected to viral detection in their bodies (IR) and heads (DIR) by RT-qPCR. Overall, a mean of 83.5% blood-fed females was recorded, considering both strains and blood conditions analysed (Additional file 2: Table S2). The mortality among the blood-fed females recorded from the experimental groups during all assays was < 4% (Additional file 2: Table S2). In females fed uninfected blood DENV-2 was not detected. In females fed infected blood and collected immediately after the oral infection, between 80 and 100% were positive for DENV-2 for both strains, proving that females ingested the infected blood (Additional file 3: Table S3). At the other time points, 180 RNA samples from females of each strain derived from three independent assays were analysed to assess the presence of virus (Additional file 3: Table S3). The statistical analysis of data from those three assays performed (Additional file 3: Table S3) showed no differences; next, they were combined to represent the infection and dissemination profiles of these strains (Fig. 1, Additional file 4: Table S4). DENV-2 was found in body samples from both strains at each time point assessed (7, 14 and 21 dpi), with infection rates that were 35, 36.7 and 53.3% for RecBti against 36.7, 40 and 45.0% for RecL samples, which did not differ significantly (Chi-square: χ^2^ = 0, *df* = 1, *P* = 1.000, χ^2^ = 0.03, *df* = 1, *P* = 0.851 and χ^2^ = 0.53, *df* = 1, *P* = 0.465, respectively) (Fig. 1a, Additional file 4: Table S4). DENV-2 was also detected in head samples from females whose body samples were positive from both strains and at all time points analysed, demonstrating virus dissemination in that tissue. The dissemination increased over time, rising from 27% to > 70% positive samples, for both strains (Fig. 1b). The dissemination in RecBti after 7 dpi (41%) and 14 dpi (82.6%) was higher than that recorded for RecL, which was 27.3% and 66.7%, respectively; however, these values were statistically similar (Fisher’s exact test: *P* = 0.511, OR = 0.62, 95% CI 0.13–2.92; *P* = 0.317, OR = 0.43, 95% CI 0.07–1.96, respectively) (Fig. 1b, Additional file 4: Table S4).

**Fig. 2:**
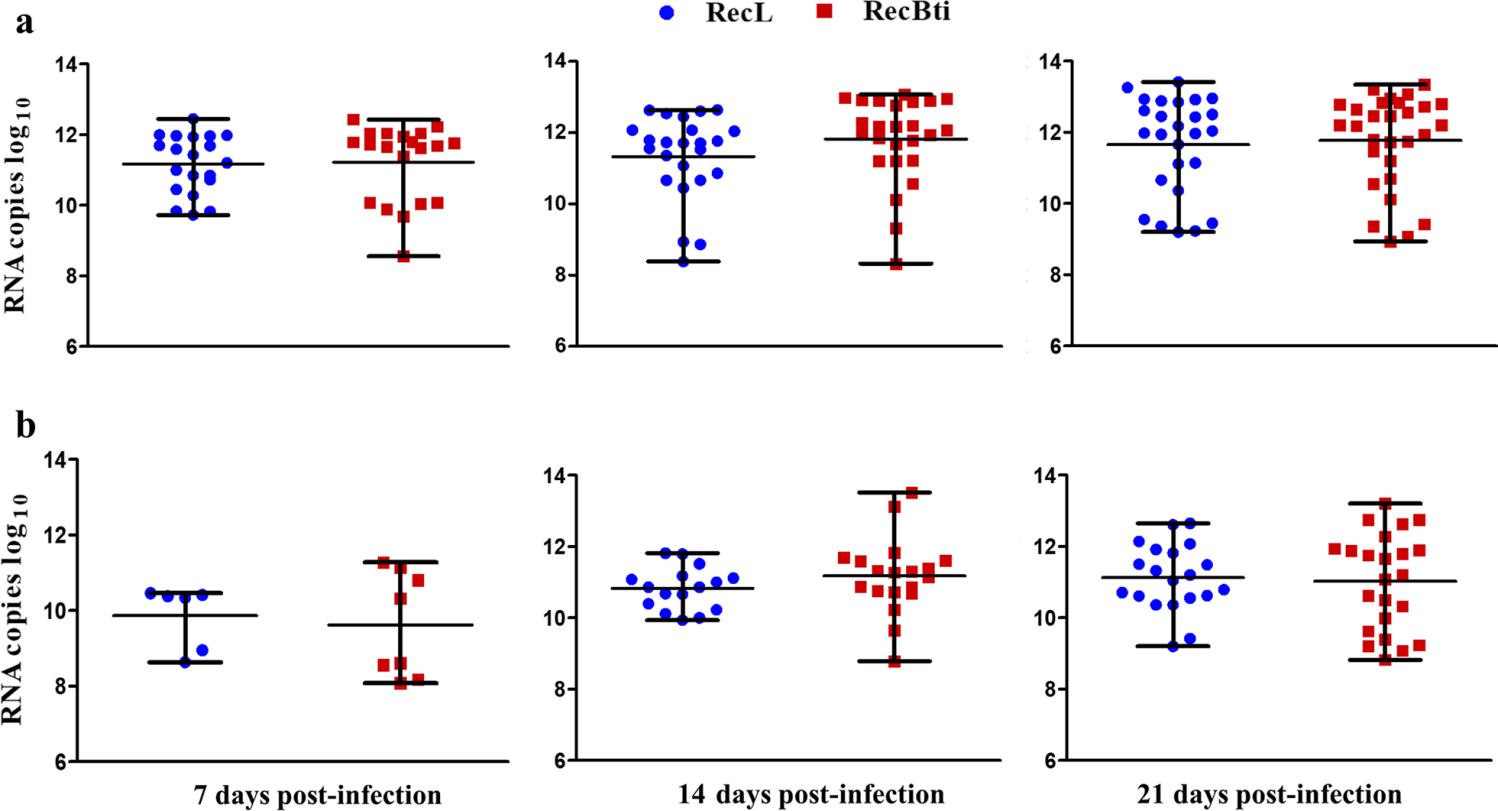
Quantitation of DENV-2 RNA in *Aedes aegypti* females from RecBti and RecL strains. **a** Viral RNA in body samples. **b** Viral RNA in head samples. Experimental points represent positive individuals from three independent assays. According Kruskal-Wallis H-test, the percentage between mosquito strains is not significantly different for *P* > 0.05

Viral quantification of DENV-2 in females samples varied from 3.6 × 10^8^ to 2.7 × 10^13^ log_10_ RNA copies. The quantification of viral copies in the body (Fig. 2a) and head (Fig. 2b) was similar between RecBti and RecL females, which showed variability within samples among time points. DENV-2 copies increased particularly in head samples from 7 to 14 dpi for both strains (Fig. 2b). Nevertheless, no significant differences regarding the quantification of viral copies between strains were observed at any time point. Datasets from these assays showed that females of both strains were susceptible to DENV-2, and although an increased dissemination in RecBti females was recorded at two time points, these results were statistically similar.

### Mosquito susceptibility to ZIKV

**Fig. 3:**
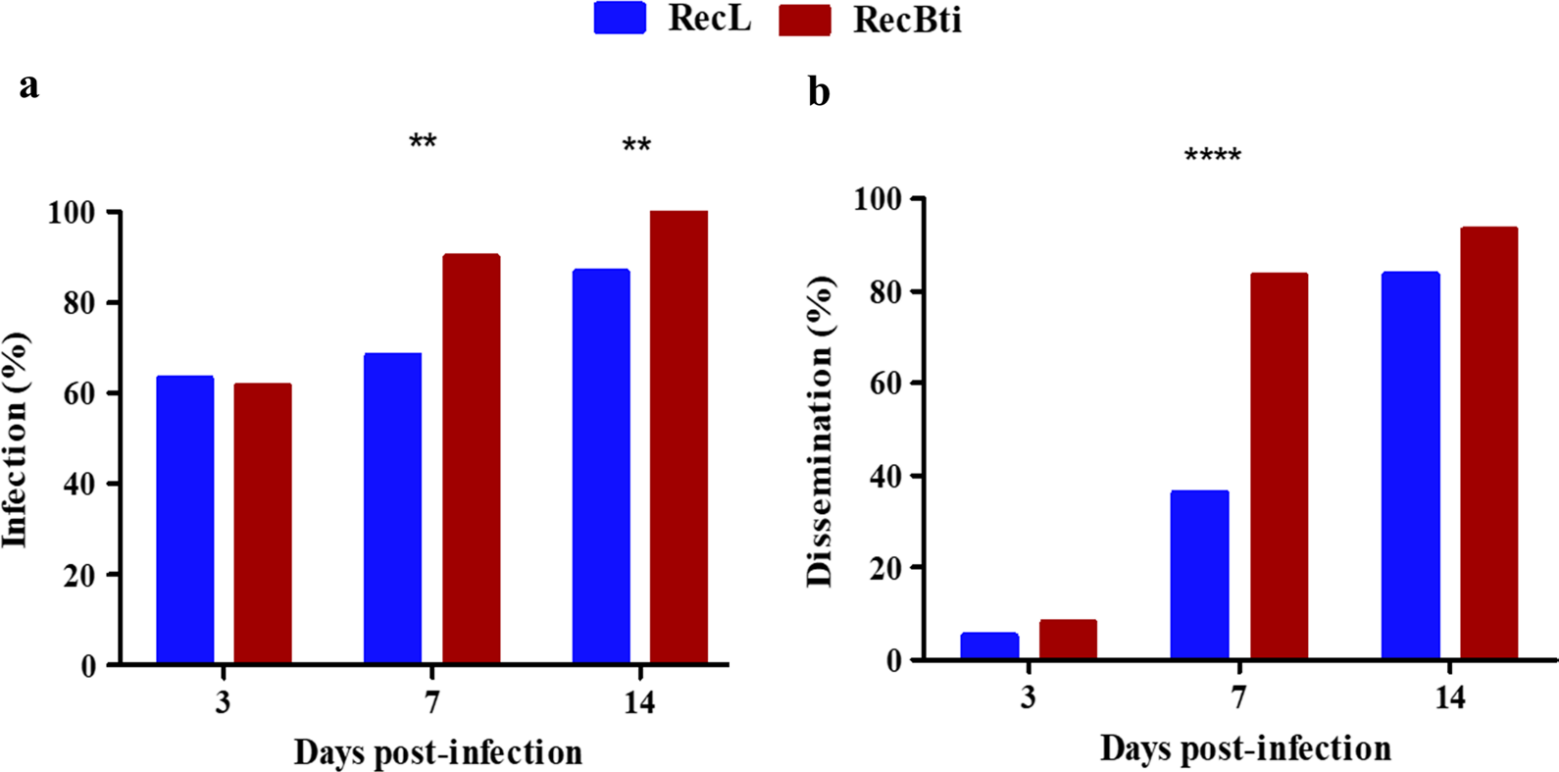
Detection of ZIKV RNA in *Aedes aegypti* females from RecBti and RecL strains. **a** Infection rate. **b** Dissemination rate. Each experimental point was based on a sample of 60 individuals analysed from three independent assays. Each column represents positive samples in absolute numbers. Asterisks indicate significant differences between values (**P ≤ 0.006; ****P < 0.0001), according Chi-square and Fisher’s exact tests

For ZIKV infection assays, a mean of 89.5% engorged females from both strains and conditions was recorded (Additional file 2: Table S2). Samples from females engorged with uninfected blood were negative for the presence of ZIKV, as expected. The mortality in all experimental groups was negligible (Additional file 2: Table S2). The presence of ZIKV RNA copies assessed immediately after oral infection was 100% in samples of both strains (Additional file 3: Table S3). Data from the three ZIKV assays performed (Additional file 3: Table S3) were combined for this analysis (Fig. 3, Additional file 4: Table S4). The data showed that both strains were more susceptible to infection using ZIKV than the DENV-2 samples tested in this study, since approximately 60% of body samples were infected at 3 dpi. The comparison of ZIKV infection between the *Ae. aegypti* strains did not show a significant difference at this time point (Fig. 3a). Infection rates after 7 and 14 dpi reached 90–100% for RecBti and were significantly higher (Chi-square: χ^2^ = 7.27, *df* = 1, *P* = 0.006 and χ^2^ = 7.69, *df* = 1, *P* = 0.005, respectively) than the 68.3% and 86.7% detected for RecL (Fig. 3a, Additional file 4: Table S4). ZIKV dissemination to head samples was more pronounced at 7 and 14 dpi (Fig. 3b). An increasing dissemination pattern throughout the time points was observed for RecBti (8.1–93.3%) and RecL (5.3–86.3%) samples, which was statistically higher for RecBti at 7 dpi (Fisher’s exact test: *P* < 0.0001, OR = 0.11, 95% CI 0.03–0.32) (Fig. 3b, Additional file 4: Table S4).

**Fig. 4:**
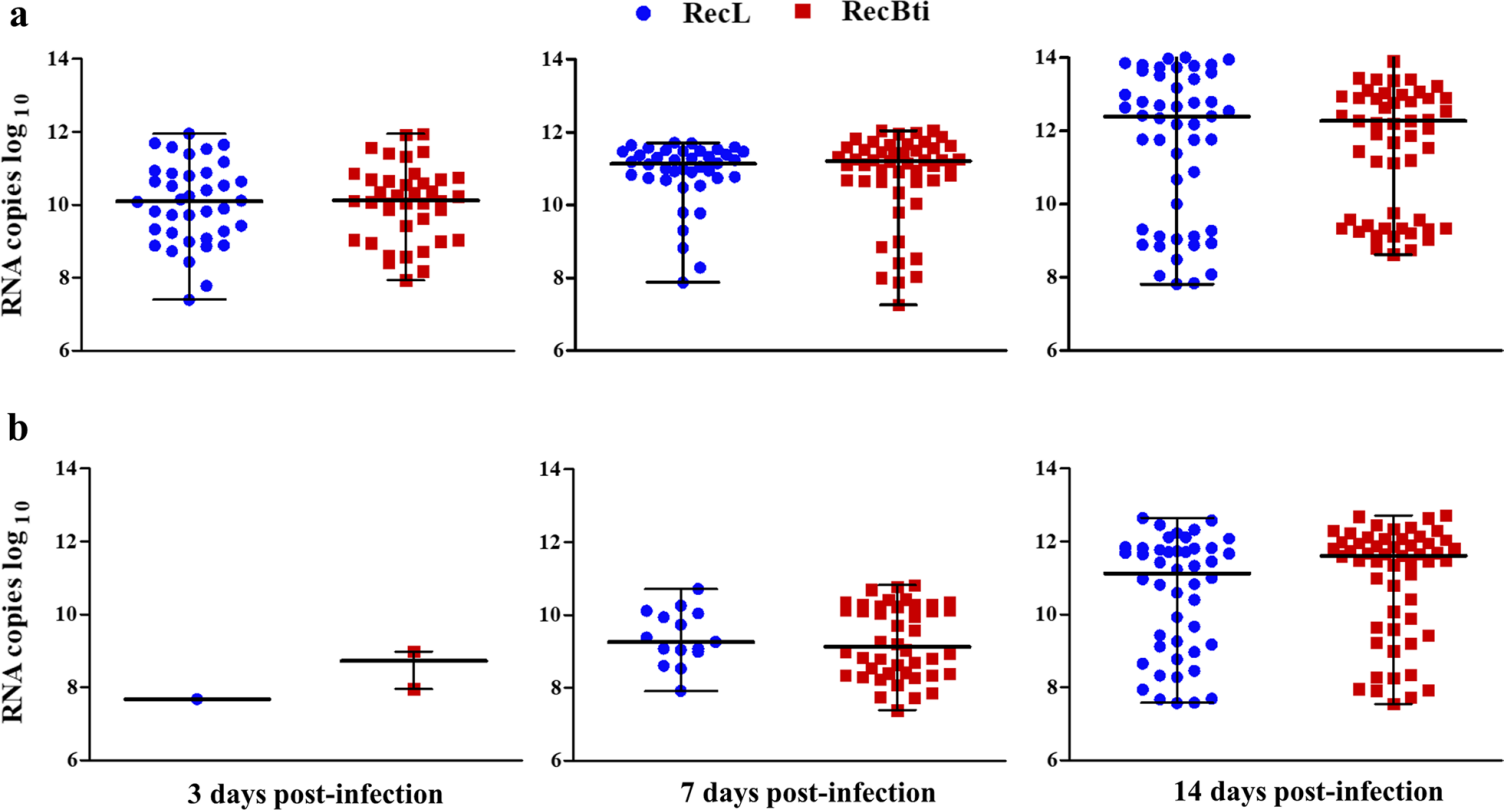
Quantitation of ZIKV RNA in *Aedes aegypti* females from RecBti and RecL strains. **a** Viral RNA in body samples. **b** Viral RNA in head samples. Experimental points represent positive individuals from three independent assays. The percentage between mosquito strains is not significantly different, for *P* > 0.05, according Kruskal-Wallis H-test

The quantification of ZIKV RNA determined in head and body samples from females that were found to be positive for the virus showed a number of RNA copies ranging from 1.8 × 10^7^ to 3.5 × 10^14^ log_10_ (Fig. 4). The number of RNA copies in the bodies of RecL and RecBti increased gradually over time, but no significant difference between strains was detected (Fig. 4a). In head samples, RNA copies increased primarily between 7 and 14 dpi and remained similar between the RecBti and RecL strains (Fig. 4b). Overall, RecBti females exhibited higher infection and dissemination rates for ZIKV than did RecL females, although the viral quantification in the positive samples from both strains was similar.

## Discussion

The prolonged exposure of *Ae. aegypti* larvae to Bti over 29 generations had no impact on the life traits analysed from immature and adult phases, and this finding is compatible with the susceptibility status of this strain to Bti [33]. It is worth noting that RecBti strain was subjected to strong and continuous exposure of all larvae from each generation to the Bti crystals [33]. Therefore, the lack of resistance was not due to a low selection pressure. Biological costs have often been associated with significant levels of resistance to chemical insecticides due to the requirement of allocating energetic resources to ensure this phenotype [58–60]. However, the reduction in insect fitness associated with resistance or exposure to bacterial-based larvicides warrants further study, since contrasting results have been reported for *Culex quinquefasciatus* strains with a high resistance ratio (RR_50_ > 5,000-fold) to *Lysinibacillus sphaericus*-based larvicides, which exhibit only discrete alterations or no biological costs [61, 62]. An *Ae. aegypti* strain exposed to Bti for 22 generations without resistance to the Bti crystal but with resistance to individual Bti toxins (Cry4Aa RR_50_ = 35, Cry4Ba RR_50_ = 11) showed a reduction in fertility rates, larval viability and increased larval development time, while adult size, sex ratio, hatching time, longevity and survival were not changed [63]. Similarly, a *Culex pipiens* strain exposed to Bti for 20 generations and still susceptible to this agent (RR_50_ = 2.7) showed a reduction in its fertility rate, while its longevity and time of blood-meal digestion were not altered [64]. Other studies that investigated the effects of sublethal doses of Bti on adult traits from susceptible strains of *Ae. aegypti* and *Anopheles coluzzii,* have noted both advantages and reductions in different biological parameters evaluated [65–67].

Differences recorded among those studies may be attributable to variations in the genetic background of mosquito species and viral strains, besides selection and rearing procedures. The assessment of insect fitness in response to exposure or resistance to *B. thuringiensis* toxins, in general, can be based on a large number of parameters [68]; in addition, variations in the adopted methodologies can lead to different results [43]. Attention should also be directed to certain specific biological mosquito features, such as protandry, that require suitable conditions for enabling the eclosion of larvae that will develop into males and females and their emergence [69]. Optimal conditions for immature development and adult maintenance are also critical for mosquito development. Therefore, under the controlled conditions of this study, *Ae. aegypti* from the RecBti strain did not exhibit changes in a number of major life traits analysed; however, other biological parameters might be affected, such as vector competence patterns.

Vector competence is the intrinsic ability of an insect to be infected with a pathogen and transmit it to another host. Few studies have investigated this feature in mosquito strains exposed to Bti, particularly after a long-term exposure period. In our study, *Ae. aegypti* RecBti females were determined to be susceptible to DENV-2 and ZIKV infection, as shown for the RecL reference strain previously assessed [50, 51]. Females from both strains were more susceptible to ZIKV than DENV, and this greater permissiveness of *Aedes* sp. to ZIKV agrees with the findings of other studies [50, 51, 70–72]. The comparison between these *Ae. aegypti* strains showed that RecBti females had significantly higher infection (at 7 and 14 dpi) and dissemination rates (at 7 dpi) for ZIKV than did the reference RecL strain at some time points. Notably, an increased dissemination in RecBti females was also observed for DENV-2, but this parameter was not statistically different that of from RecL females. Quantification of viral copies was similar for both arboviruses in the mosquito strains; therefore, the increased susceptibility to ZIKV was related to the greater infection and dissemination rates only [51, 53, 73, 74].

To the best of our knowledge, our study is the first to report the effect of chronic exposure of *Ae. aegypti* to Bti during their larval phase (29 preceding generations) on the susceptibility of females for ZIKV. Most studies have investigated the arbovirus susceptibility of females exposed to sublethal doses of Bti during the larval phase but without a history of previous Bti exposure. Moltini-Conclois et al. [75] assessed the susceptibility of *Ae. aegypti* to DENV-1 and CHIKV in females from a susceptible strain, a Cry4Aa-resistant strain (RR = 1018) and a composite Bti-selected strain (susceptible to Bti with variable resistance from 5- to 14-fold to individual toxins). No alterations were observed for CHIKV, and enhanced susceptibility for DENV-1 was detected for Cry4A-resistant and Bti-selected females treated with sublethal Bti doses, similar to larvae. However, this effect was not observed in females that were not treated during their larval phase [75]. This finding suggests that increased DENV-1 susceptibility could be related to recent Bti larval exposure rather than to the previous status of Bti susceptibility of the strains tested. Another study showed no changes in DENV-1 susceptibility in *Ae. aegypti* females exposed as larvae to sublethal Bti doses [67]. Carlson et al. [76] investigated the carry-over effect of *Ae. aegypti* on susceptibility to ZIKV and DENV after larval and/or adult exposure to *Enterobacter ludwiggi* and to a *B. thuringiensis* (ATCC #35646) crystal-producing strain that was not specified as Bti. Significant alterations were seen in females exposed to *E. ludwiggi* during either larval (lower DENV-2 infection) or adult phases (higher ZIKV infection). Females originating from *B. thuringiensis*-treated larvae exhibited no alteration. Contrary to other published findings, our study showed an increased vector susceptibility to ZIKV not associated with an induced response related to the Bti exposure of tested individuals during their larval phase but rather a constitutive profile associated with the background of bacterial exposure during the 29 preceding generations.

The enhanced viral susceptibility of RecBti females might be further investigated, considering the role of the immune system and microbiota in modulating such responses, as reviewed by Caragata et al. [77], and few studies have investigated the role played by Bti in this process. An example was an *Ae. aegypti* strain exposed for 18 generations (RR_50_ = 2) that exhibited reduced expression of genes from the Toll signalling pathway and genes coding antimicrobial peptides [78]. The Toll pathway is widely known in the defence of *Ae. aegypti* to DENV-2 [79] and ZIKV [80], as well as JAK/STAT [81], since they can activate effector molecules that limit viral spread. *Aedes aegypti* antimicrobial peptides, such as defensins and cecropins, with antiviral action have also been reported [82]. The microbiota in the mosquito larval environment and in the larval midgut [74, 83] can provoke changes in adult traits, including vector competence [74, 84]. Therefore, Bti, as a bacterial pathogen present in the aquatic environment and ingested by mosquito larvae [11], might play an important role in these microbiota. Indeed, *Ae. aegypti* larvae from a laboratory-susceptible strain exposed to Bti for 25 h exhibited a reduction in the diversity of bacteria [85]. As previously described, the RecBti and RecL strains compared in our study derived from the same geographic area; they were maintained under identical and controlled conditions of insectary, except for the strong and continuous condition of Bti exposure imposed to the RecBti larvae. Although the reduced susceptibility to the arbovirus tested was found to be associated to this specific condition, those strains were not established in parallel; therefore, it is not possible to discard other factors that could have an influence on those findings.

It is important to note that the association of larval exposure to Bti and a higher susceptibility of *Ae. aegypti* females for the virus investigated in our study cannot be extrapolated to the epidemiological level. This finding is observed because the process of virus transmission to humans is complex and depends on multiple factors. Some of these properties are very powerful, such as mosquito density and longevity [86], features of viral strains and the susceptibility status of the human population, which might overcome the consequences of reduced or increased vector competence. Previous investigations showed that the status of mosquito susceptibility, per se, displays a wide range of variations among populations [53, 57, 87–89] in addition to other conditions that also modulate arbovirus transmission.

## Conclusions

Our studies show that the RecBti *Ae. aegypti* strain exposed to Bti for 29 preceding generations exhibited no altered life traits, but females showed an increase in the susceptibility status to ZIKV, associated with a greater dissemination. This feature was not induced by larval exposure of tested females, as shown by other studies, but it was rather a response associated with long-term and continuous exposure to Bti. This work paves the way for further research investigating other mechanisms and phenotypes associated with chronic exposure to this control agent, which is of strategic importance for its rational utilization.

## Supplementary Information

**Additional file 1: Table S1.** Primers used for the detection and quantitation of DENV-2 and ZIKV.

**Additional file 2: Table S2.** Summary of the artificial blood-meal assays offered to *Aedes aegypti* females from RecBti and RecL strains using uninfected and infected DENV-2 and ZIKV samples.

**Additional file 3: Table S3.** Infection and dissemination rates of DENV-2 and ZIKV in *Aedes aegypti* females from RecBti and RecL strains. Data from three independent assays.

**Additional file 4: Table S4.** Infection and dissemination rates of DENV-2 and ZIKV in *Aedes aegypti* females from RecBti and RecL strains. Combined data from three independent assays (see Additional file 3: Table S3).

## Data Availability

Data supporting the conclusions are included within the article and the additional files.

## Abbreviations

DENV: Dengue virus
CHIKV: Chikungunya virus
ZIKV: Zika virus
Bti: *Bacillus thuringiensis* svar. *israelensis*
RR: Resistance ratio
LC: Lethal concentration
DENV-2: Dengue virus serotype 2
RNA: Ribonucleic acid
Ct: Cycle threshold
dpi: Day(s) post-infection
TCID_50_: Tissue culture infection dose at 50%
PFU: Plaque-forming unit
RT-qPCR: Real-time quantitative polymerase chain reaction
IR: Infection rate
DIR: Disseminated infection rate
