# Supplementary material for: Aedes aegypti continuously exposed to Bacillus thuringiensis svar. israelensis does not exhibit changes in life traits but displays increased susceptibility for Zika virus
Source: Parasit Vectors. 2021 Jul 28;14:379. doi: 10.1186/s13071-021-04880-6 (PMC8317411; doi:10.1186/s13071-021-04880-6)
Supplement: Supplementary file 1 — Additional file 1: Table S1. Primers used for the detection and quantitation of DENV-2 and ZIKV. [file 13071_2021_4880_MOESM1_ESM.docx]

**Additional file 1: Table S1.** Primers and probe used for the detection and quantitation of DENV-2 and ZIKV.

| Target | Primer (5’-3’) | Amplicon ^a^ |
| --- | --- | --- |
| DENV-2 ^b^ | Forward: GGAAGGAGAAGGACTGCACA | 104 |
|  | Reverse: ATTCTTGTGTCCCATCCTGCT |  |
| ZIKV ^c^ | Forward: CCGCTGCCCAACACAAG | 76 |
|  | Reverse: CCACTAACGTTCTTTTGCAGACAT |  |
|  | Probe: AGCCTACCTTGACAAGCAGTCAGACACTCAA |  |

^a^ Size in base pairs.

^b^ Primers amplify a region of the DENV non-structural protein 5 (NS5) [1].

^c^ Primers amplify a region of the ZIKV envelope protein [2].

**References**

1. Kong YY, Thay CH, Tin TC, Devi S. Rapid detection, serotyping and quantitation of dengue viruses by TaqMan real-time one-step RT-PCR. J Virol Methods. 2006;138 1-2:123-30; doi: 10.1016/j.jviromet.2006.08.003.

2. Lanciotti RS, Kosoy OL, Laven JJ, Velez JO, Lambert AJ, Johnson AJ, et al. Genetic and serologic properties of Zika virus associated with an epidemic, Yap State, Micronesia, 2007. Emerg Infect Dis. 2008;14 8:1232-9; doi: 10.3201/eid1408.080287.
