# Supplementary material for: Aedes aegypti continuously exposed to Bacillus thuringiensis svar. israelensis does not exhibit changes in life traits but displays increased susceptibility for Zika virus
Source: Parasit Vectors. 2021 Jul 28;14:379. doi: 10.1186/s13071-021-04880-6 (PMC8317411; doi:10.1186/s13071-021-04880-6)
Supplement: Supplementary file 2 — Additional file 2: Table S2. Summary of the artificial blood-meal assays offered to Aedes aegypti females from RecBti and RecL strains using uninfected and infected DENV-2 and ZIKV samples. [file 13071_2021_4880_MOESM2_ESM.docx]

**Additional file 2: Table S2.** Summary of the artificial blood meal assays offered to *Aedes aegypti* females from RecBti and RecL strains using uninfected and infected DENV-2 and ZIKV samples.

| Paremeters | Infected | | | | |  | Not infected | | | | |
| --- | --- | --- | --- | --- | --- | --- | --- | --- | --- | --- | --- |
|  | RecBti | | RecL | | Statistics |  | RecBti | | RecL | | Statistics |
| DENV-2 | *n* | % | *n* | % | Student’s t-test |  | *n* | % | *n* | % | Student’s t-test |
| Blood-fed | 312 | 84.3 | 332 | 89.7 | *t*_(4)_ = 2.77, *P* = 0.67 |  | 77 | 85.6 | 68 | 75.6 | *t*_(4)_ = 2.77, *P* = 0.03^a^ |
| Unfed | 58 | 15.7 | 38 | 10.3 | *t*_(4)_ = 2.77, *P* = 0.42 |  | 13 | 14.4 | 22 | 24.4 | *t*_(4)_ = 2.77, *P* = 0.03^a^ |
| Mortality (blood-fed) | 6 | 1.9 | 3 | 0.9 | *t*_(4)_ = 2.77, *P* = 0.48 |  | 3 | 3.9 | 0 | 0 | *t*_(4)_ = 2.77, *P* = 0.22 |
| ZIKV |  |  |  |  |  |  |  |  |  |  |  |
| Blood-fed | 339 | 95.5 | 321 | 90.4 | *t*_(4)_ = 2.77, *P* = 0.33 |  | 75 | 83.3 | 76 | 84.4 | *t*_(4)_ = 2.77, *P* = 0.85 |
| Unfed | 16 | 4.5 | 34 | 9.6 | *t*_(4)_ = 2.77, *P* = 0.08 |  | 15 | 16.7 | 14 | 15.6 | *t*_(4)_ = 2.77, *P* = 0.85 |
| Mortality (blood-fed) | 0 | 0 | 3 | 1 | *t*_(4)_ = 2.77, *P* = 0.18 |  | 0 | 0 | 1 | 0.3 | *t*_(4)_ = 2.77, *P* = 0.42 |

^a^ *P* ≤ 0.05 is significantly different, for Student’s t-test
