# Supplementary material for: Aedes aegypti continuously exposed to Bacillus thuringiensis svar. israelensis does not exhibit changes in life traits but displays increased susceptibility for Zika virus
Source: Parasit Vectors. 2021 Jul 28;14:379. doi: 10.1186/s13071-021-04880-6 (PMC8317411; doi:10.1186/s13071-021-04880-6)
Supplement: Supplementary file 3 — Additional file 3: Table S3. Infection and dissemination rates of DENV-2 and ZIKV in Aedes aegypti females from RecBti and RecL strains. Data from three independent assays. [file 13071_2021_4880_MOESM3_ESM.docx]

**Additional file 3: Table S3.** Infection and dissemination rates of DENV-2 and ZIKV in *Aedes aegypti* females from RecBti and RecL strains. Data from three independent assays.

| Viruses | Dpi ^a^ | *n* | Infection | | | | |  | Dissemination | | | | |
| --- | --- | --- | --- | --- | --- | --- | --- | --- | --- | --- | --- | --- | --- |
|  |  |  | RecBti | | RecL | |  |  | RecBti | | RecL | |  |
|  |  |  | + ^b^ | % | + ^b^ | % | Fisher’s exact tests ^c^ |  | + ^b^ | % | + ^b^ | % | Fisher’s exact tests ^c^ |
| DENV-2 |  |  |  |  |  |  |  |  |  |  |  |  |  |
| 1 | 0 | 5 | 4 | 80 | 5 | 100 | *P* = 1.00, OR: 0.91, CI: 0.38-2.14 |  | - | - | - | - | - |
|  | 7 | 20 | 5 | 25 | 2 | 10 | *P* = 0.41, OR: 0.34, CI: 0.03-2.47 |  | 1 | 25 | 0 | 0 | *P* = 1.00, OR: 0.72, CI: 0.0-15.37 |
|  | 14 | 20 | 3 | 15 | 5 | 25 | *P* = 0.69, OR: 1.86, CI: 0.30-14.1 |  | 2 | 60 | 3 | 45 | *P* = 1.00, OR: 0.77, CI: 0.01-27.72 |
|  | 21 | 20 | 6 | 30 | 6 | 30 | *P* = 1.00, OR: 1.00, CI: 0.21-4.79 |  | 5 | 70 | 3 | 35 | *P* = 0.54, OR: 0.23, CI: 0.0-4.50 |
| 2 | 0 | 5 | 5 | 100 | 5 | 100 | *P* = 1.00, OR: 0.65, CI: 0.31-1.37 |  | - | - | - | - | - |
|  | 7 | 20 | 9 | 45 | 8 | 40 | *P* = 1.00, OR: 1.00, CI: 0.24-4.13 |  | 4 | 38 | 4 | 38 | *P* = 1.00, OR: 0.73, CI: 0.01-23.35 |
|  | 14 | 20 | 8 | 40 | 4 | 20 | *P* = 0.30, OR: 0.38, CI: 0.07-1.85 |  | 8 | 100 | 2 | 50 | *P* = 0.09, OR: 0.05, CI: 0.0-2.37 |
|  | 21 | 20 | 10 | 50 | 8 | 40 | *P* = 0.75, OR: 0.67, CI: 0.15-2.76 |  | 5 | 50 | 7 | 87 | *P* = 0.15, OR: 6.27, CI: 0.48-376.30 |
| 3 | 0 | 5 | 5 | 100 | 5 | 100 | *P* = 1.00, OR: 1.33, CI: 0.63-2.81 |  | - | - | - | - | - |
|  | 7 | 20 | 8 | 40 | 11 | 55 | *P* = 0.53, OR: 1.80, CI: 0.44-7.72 |  | 3 | 43 | 2 | 20 | *P* = 0.60, OR: 0.39, CI: 0.02-4.67 |
|  | 14 | 20 | 11 | 55 | 15 | 75 | *P* = 0.32, OR: 2.39, CI: 0.53-11.9 |  | 9 | 94 | 11 | 78 | *P* = 1.00, OR: 0.92, CI: 0.10-7.11 |
|  | 21 | 20 | 16 | 80 | 13 | 65 | *P* = 0.48, OR: 0.47, CI: 0.08-2.36 |  | 13 | 98 | 10 | 87 | *P* = 1.00, OR: 0.78, CI: 0.08-7.10 |
| ZIKV |  |  |  |  |  |  |  |  |  |  |  |  |  |
| 1 | 0 | 5 | 5 | 100 | 5 | 100 | *P* = 1.00, OR: 0.67, CI: 0.08-5.34 |  | - | - | - | - | - |
|  | 3 | 20 | 10 | 50 | 12 | 60 | *P* = 1.00, OR: 1.00, CI: 0.24-4.13 |  | 0 | 0 | 0 | 0 | *P* = 1.00, OR: 0.89, CI: 0.10-9.24 |
|  | 7 | 20 | 20 | 100 | 8 | 40 | *P* < 0.0001^c^, OR: 0.0, CI: 0.0-0.19 |  | 16 | 88 | 1 | 12.5 | *P* = 0.001^c^, OR: 0.04, CI: 0.0-0.45 |
|  | 14 | 20 | 20 | 100 | 15 | 75 | *P* = 0.02^c^, OR: 0.0, CI: 0.0-0.72 |  | 17 | 90 | 9 | 61. | *P* = 0.09, OR: 0.21, CI: 0.02-1.59 |
| 2 | 0 | 5 | 5 | 100 | 5 | 100 | *P* = 1.00, OR: 0.89, CI: 0.22-7.28 |  | - | - | - | - | - |
|  | 3 | 20 | 11 | 55 | 11 | 55 | *P* = 1.00, OR: 1.00, CI: 0.24-4.35 |  | 0 | 0 | 0 | 0 | *P* = 1.00, OR: 0.84, CI: 0.10-9.25 |
|  | 7 | 20 | 14 | 70 | 15 | 75 | *P* = 1.00, OR: 1.27, CI: 0.25-6.62 |  | 12 | 78.1 | 5 | 33.5 | *P* = 0.02^c^, OR: 0.14, CI: 0.01-0.90 |
|  | 14 | 20 | 20 | 100 | 17 | 85 | *P* = 0.23, OR: 0.0, CI: 0.0-2.34 |  | 20 | 100 | 18 | 94.7 | *P* = 0.49, OR: 0.0, CI: 0.0-37.05 |
| 3 | 0 | 5 | 5 | 100 | 5 | 100 | *P* = 1.00, OR: 0.75, CI: 0.34-9.15 |  | - | - | - | - | - |
|  | 3 | 20 | 16 | 80 | 15 | 75 | *P* = 1.00, OR: 0.76, CI: 0.12-4.27 |  | 2 | 12 | 1 | 5 | *P* = 0.65, OR: 1.72, CI: 0.16-23.84 |
|  | 7 | 20 | 20 | 100 | 18 | 90 | *P* = 0.49, OR: 0.0, CI: 0.0-5.28 |  | 17 | 85 | 9 | 50 | *P* = 0.01^c^, OR: 0.11, CI: 0.01-0.74 |
|  | 14 | 20 | 20 | 100 | 20 | 100 | *P* = 1.00, OR: 0.0, CI: 0.15-9.56 |  | 18 | 90 | 19 | 95 | *P* = 1.00, OR: 2.07, CI: 0.09-130.88 |

^a^ Day(s) post-infection

^b^ Positive samples

^c^ *P* ≤ 0.05 is significantly different, for Fisher’s exact tests
