# Supplementary material for: Aedes aegypti continuously exposed to Bacillus thuringiensis svar. israelensis does not exhibit changes in life traits but displays increased susceptibility for Zika virus
Source: Parasit Vectors. 2021 Jul 28;14:379. doi: 10.1186/s13071-021-04880-6 (PMC8317411; doi:10.1186/s13071-021-04880-6)
Supplement: Supplementary file 4 — Additional file 4: Table S4. Infection and dissemination rates of DENV-2 and ZIKV in Aedes aegypti females from RecBti and RecL strains. Combined data from three independent assays (see Additional file 3: Table S3). [file 13071_2021_4880_MOESM4_ESM.docx]

**Additional file 4: Table S4.** Infection and dissemination rates of DENV-2 and ZIKV in *Aedes aegypti* females from RecBti and RecL strains. Combined data from three independent assays (see Additional file 3: Table S3).

| Viruses | Dpi ^a^ | *n* | Infection | | | | |  | Dissemination | | | | |
| --- | --- | --- | --- | --- | --- | --- | --- | --- | --- | --- | --- | --- | --- |
|  |  |  | RecBti | | RecL | | Statistics |  | RecBti | | RecL | | Statistics |
|  |  |  | + ^b^ | % | + ^b^ | % | Chi-square test |  | + ^b^ | % | + ^b^ | % | Fisher’s exact tests |
| DENV-2 | 0 | 15 | 14 | 93.3 | 15 | 100 | χ^2^ = 0.88, *df* = 1, *P* = 0.42 |  | - | - | - | - | *-* |
|  | 7 | 60 | 21 | 35.0 | 22 | 36.7 | χ^2^ = 0.0, *df* = 1, *P* = 1.00 |  | 8 | 41.0 | 6 | 27.3 | *P* = 0.51, OR :0.62, CI: 0.12-2.92 |
|  | 14 | 60 | 22 | 36.7 | 24 | 40.0 | χ^2^ = 0.03, *df* = 1, *P* = 0.85 |  | 19 | 82.6 | 16 | 66.7 | *P* = 0.32, OR: 0.42, CI: 0.08-1.96 |
|  | 21 | 60 | 32 | 53.3 | 27 | 45.0 | χ^2^ = 0.53, *df* = 1, *P* = 0.46 |  | 23 | 71.8 | 20 | 74.0 | *P* = 1.00, OR: 1.11, CI: 0.30-4.23 |
| ZIKV | 0 | 15 | 15 | 100 | 15 | 100 | χ^2^ = 0.0, *df* = 1, *P* = 1.00 |  | - | - | - | - | - |
|  | 3 | 60 | 37 | 61.7 | 38 | 63.3 | χ^2^ = 0.0, *df* = 1, *P* = 1.00 |  | 2 | 8.1 | 1 | 5.3 | *P* = 0.67, OR: 1.58, CI: 0.17-19.89 |
|  | 7 | 60 | 54 | 90.0 | 41 | 68.3 | χ^2^ = 7.27, *df* = 1, *P* = 0.006^c^ |  | 48 | 83.3 | 15 | 36.0 | *P* < 0.001^c^ OR: 0.11, CI: 0.03-0.32 |
|  | 14 | 60 | 60 | 100 | 52 | 86.7 | χ^2^ = 7.69, *df* = 1, *P* = 0.005^c^ |  | 55 | 93.3 | 46 | 83.6 | *P* = 0.34, OR: 0.47, CI: 0.09-1.99 |

^a^ Day(s) post-infection

^b^ Positive samples

^c^ *P* ≤ 0.05 is significantly different, for Chi-square test or Fisher’s exact test
